# Conceptual Evolution and Current Approach to Spitz Tumors

**DOI:** 10.3390/dermatopathology9020017

**Published:** 2022-04-26

**Authors:** Carmelo Urso, Vincenzo De Giorgi, Daniela Massi

**Affiliations:** 1 Dermatopathology Study Center of Florence, 50129 Florence, Italy; cylaur@tin.it; 2 Dermatology Unit, Azienda USL Toscana Centro, and Section of Dermatology, Department of Health Sciences, University of Florence, 50122 Florence, Italy; vincenzo.degiorgi@unifi.it; 3 Section of Anatomic Pathology, Department of Health Sciences, University of Florence, Viale Pieraccini 6, 50139 Florence, Italy

**Keywords:** spitz nevus, atypical spitz tumor, spitz melanoma, dermatopathology

## Abstract

Over the past several decades, the study of Spitz neoplasms has flourished, with expanded conceptualization and refined terminology, providing a framework for the assessment and classification of Spitz nevi, atypical Spitz Tumors, and Spitz melanoma. Cancer genomics have generated concepts such as driver and passenger genes and clonal evolution, which can be applied to Spitz tumors. Herein, we provide a historical perspective, followed by a summary of current knowledge and clinical approaches for these challenging tumors.

In the first half of the last century, melanoma was described as a pigmented and often ulcerated malignant tumor with a very poor prognosis [1]. Clinical observations suggested that melanomas of infancy and childhood, although histologically indistinguishable from adult melanomas, seldom developed metastases [2]. In children, such tumors (prepubertal melanomas) histologically resembled malignant melanomas but did not behave as such [3]. The distinctive histologic features of these melanomas with a relatively favorable prognosis, occurring in childhood, were described (juvenile melanomas) [4]. Soon after, however, opinions rapidly changed. Because of their benign clinical behavior, juvenile melanomas were classified among benign nevi [5], and a set of histological criteria was provided: relatively superficial lesion; subepidermal edema and teleangiectasias; spherical or spindle cells; large cells with myogeneous-appearing cytoplasm; superficial giant cells; abrupt transition between junctional cells and intact epidermis; sparsity of pigmentation [6]. Because of their benign nature and occurrence in adults, juvenile melanomas were renamed *spindle cell nevi* or *spindle cell* and *epithelioid cell nevi* [7]. Subsequently, a committee of Australian Pathologists proposed the term *Spitz nevus*, giving credit to the late Sophie Spitz, who died in 1956 [8].

Spitz nevus was regarded as a fully benign lesion showing atypical histological features and, therefore, resembling or mimicking melanoma [9,10]. Cases diagnosed as such, but subsequently metastasizing, were regarded as diagnostic errors [7]. However, some genuine melanomas having histologic features of Spitz nevus were also described [11]; these malignant tumors seemed to resemble or mimic Spitz nevi, in that they were simulators of a simulator. Thus, the theory of the double, mutual simulation was elaborated: Spitz nevus resembled, mimicked, or simulated melanoma, and some melanomas resembled, mimicked, or simulated Spitz nevus [12]. In this intricate context, the differential diagnosis of Spitz nevus and melanoma became an authentic puzzle, in which, in order to avoid the trap of pseudomalignancy, diagnosing as malignant a benign lesion (Spitz nevus mimicking melanoma), it was possible to fall into the worst trap of pseudo-pseudomalignancy, diagnosing as benign a malignant tumor (melanoma mimicking Spitz nevus). Not surprisingly, reproducibility in the diagnosis of Spitz nevus was rather low [13].

Years later, some lesions with the histological features of Spitz nevi with lymph node metastases were reported. Such cases, termed as *malignant Spitz nevi*, were not considered diagnostic errors, or labeled as melanomas, because their histological features were not considered sufficient to support such a diagnosis, and because they failed to show “the potential of widespread metastasis” [14]. This interpretation led to the recognition of a pathological category histologically and biologically intermediate between benign Spitz nevus and melanoma. Subsequently, such a diagnostic category was labeled as *metastasizing Spitz tumor* and *atypical Spitz nevus/tumor* [15,16]. However, an intermediate category between nevus and melanoma was not promptly and universally accepted and the relative terminology, which connected words conveying divergent concepts, was consequently criticized [17,18]. Therefore, the two different views on lesions described by Spitz were at odds with one another [19]: (1) Spitz nevus and melanoma are two distinct lesions; sometimes such a distinction can be difficult, but suitable histological criteria can and must be found [7,9,10,17,18,20]; (2) from benign Spitz nevus to melanoma there exists a spectrum of lesions, including intermediate forms (atypical Spitz nevi/tumors); histological criteria to correctly diagnose classical Spitz nevi and classical melanomas are available, but reliable histological criteria to diagnose intermediate forms seem to be lacking [11,14,15,16,21]. These two different views, however, basically shared the same perspective. Skin melanocytic lesions were then classified in either benign nevi or melanomas [22], Spitz nevus was regarded as a nevus variant [22,23,24,25], and malignant lesions displaying large epithelioid and spindle melanocytes were diagnosed as *Spitzoid melanomas* and considered special variants of melanoma [11,16,26].

Although largely accepted, this interpretation generated doubts, because Spitz nevi (and Spitzoid melanomas) were composed of a peculiar type of cell, a characteristic large epithelioid and/or spindle melanocyte with abundant cytoplasm, absent in other types of melanocytic lesions [27]. This distinctive morphologic feature suggested that lesions described by Spitz constituted a distinct class of benign and malignant tumors [28,29]. They could be properly termed as *Spitz nevi* and *Spitz melanomas* (cumulatively *Spitz tumors*), rather than *Spitzoid*, because they did not resemble, but actually were, the tumors originally described by Spitz [29]. On this basis, benign Spitz tumors, currently included among nevi, were not variants of nevi, and Spitz melanomas were not variants of melanomas. Spitz tumors were not simulators of melanoma and were not simulators at all; they simulated no other lesion but themselves [29]. This different perspective suggested that the histopathological features useful for differentiation between conventional nevus and conventional melanoma might not work properly to distinguish Spitz nevus from Spitz melanoma, because they were not the right key for the lock we wanted to open; this could help to explain why, not infrequently, they failed [29,30,31].

In more recent years, genomic studies have shown that most Spitz neoplasms harbor serine–threonine kinase mutations (HRAS, or more rarely MAP2K1) or receptor tyrosine and serine–threonine kinase fusions (ALK, ROS1, NTRK1, NTRK3, MAP3K8, more rarely BRAF, RET, MET, NTRK2, ERB4, FGFR1, MAP3K3, PRKDC) [32,33,34,35,36,37,38]. Of note, BRAF or MAP3K8-fused cases may pursue a more aggressive course compared with tyrosine kinase–fused cases, especially in the presence of TERT promoter mutations. The genomic abnormalities seem to act as driver events, initiating the oncogenic process and conferring some peculiar biological and morphological features, including the characteristic large spindle/epithelioid cells. The neoplastic pathway may start with a single driver mutation/fusion, inducing an increase in cellular proliferation, and, consequently, an increase in the probability that additional mutations occur. Supervening mutations may be ineffective or capable to modify, slightly or severely, one or more of the cell proliferation control mechanisms. If effective, they may enhance cell proliferation and increase the probability that other mutations occur [39]. Some Spitz lesions show no additional or few genomic abnormalities, whereas others harbor a high number of new mutations that contribute to promoting the oncogenic process, including CDKN2A loss, TERT-promoter, PTEN loss, and TP53 (promoting mutations). These latter chromosomal aberrations tend to ablate some tumor-suppression mechanisms and to activate further oncogenic pathways, thus contributing to confer characteristics related to the clinical behavior, such as the capacity for local invasion and metastasis. Furthermore, a fraction of Spitz tumors, in association with the driver mutation/fusion, express a variable, relatively low number of promoting mutations [39]. Therefore, in a certain moment of its history, any given Spitz lesion comes to possess a certain number of acquired pathogenic mutations, generating a corresponding proportional risk of neoplastic progression and, consequently, future possible adverse events (recurrences; local/regional/distant metastases; death). When genomic alterations are small in number, limited to the driver mutation or few more, this risk is minimal, adverse events are rare or very rare, and, clinically and histologically, there is a strong probability that the tumor will be classified as benign. When genomic alterations are numerous, including the driver and promoting mutations, the risk is high or very high, adverse events are relatively frequent, and, clinically and histologically, it is more probable that the tumor will be classified as malignant. In cases with a variable and intermediate number of acquired pathogenic promoting mutations, the risk may range between a minimum, which should be >0, and a theoretic maximum [40]; clinically and histologically, it is probable that these tumors will be classified as ambiguous or intermediate.

Based on the latest WHO Classification, Spitz tumors are considered a distinct group of melanocytic neoplasms and are acknowledged to represent one of the nine pathways (pathway 4) along which lesions progressively evolve from benign nevus to melanoma [39,41,42,43].

Spitz tumors encompass a biological spectrum of tumors with characteristic cytologic and architectural features common to all stages, with increasing morphologic atypia and genomic aberrations, from Spitz nevus to atypical Spitz tumor to Spitz melanoma (Figure 1, Figure 2 and Figure 3). Consequently, these categories appear to be more “prognostic”, rather than diagnostic [35], since a certain risk of progression is admitted in all these forms, including Spitz nevus, in which the risk of progression is estimated as minimal or very low [42]. More recently, a proposal to restrict the term “atypical Spitz tumors” to “diagnostically uncertain lesions”, and to apply the term “Spitz melanocytoma” for histologically and genetically-proven atypical Spitz tumors with CDKN2a inactivation has been made, but this distinction has not yet been universally accepted [44].

The diagnosis of Spitz tumors may be difficult on clinical, dermoscopic, and histologic grounds [31,32,33,34,35,36,37,38,39,40,41,42,43,44,45]. Clinically, they may present either as pigmented, hypopigmented, or non-pigmented lesions. Although specific dermoscopic criteria are not yet available, certain features might be helpful. Dermoscopically, they may show different features, including starburst, globular, homogeneous, pink, black pigment network, and atypical vascular patterns. In amelanotic atypical Spitz tumors, dermoscopic diagnosis may be relatively easy, due to the peculiar vessel distribution and morphology. In particular, glomerular (coiled) vessels uniformly distributed within the entire lesion without asymmetry may represent a peculiar feature that allows the differentiation from amelanotic melanoma [46].

Histologic diagnosis is currently based on microscopic characteristics (large spindle and/or epithelioid cells), possibly confirmed by an immunohistochemical or molecular evaluation of the genetic profile, including the presence of HRAS/MAP2K1 mutations and receptor tyrosine/serine–threonine kinase fusions. Importantly, Spitz tumors do not harbor GNAQ/GNA11 mutations, occurring in blue tumors, CTNNB1 mutation or APC loss, observed in deep penetrating tumors, PRKAR1A or PRKCA mutations, common in pigmented epithelioid melanocytomas, and BAP1 inactivation, characteristic of BAP1-inactivated melanocytic tumors [36,38] (Table 1). By definition, Spitz melanoma has a kinase-fusion or HRAS aberration, while it does not carry the driver mutation of a conventional melanoma, such as BRAF/NRAS or NF1 mutation. In contrast, if BRAF/NRAS or NF1 mutations are demonstrated in association with spindle and/or epithelioid morphology, the tumor should be classified as “Spitzoid melanoma”. At CGH analysis, lesions classifiable as Spitz nevi generally show isolated gains of 7p and 11p, and, sometimes, tetraploidy, whereas lesions classifiable as atypical Spitz tumors or Spitz melanomas generally show more than 1 chromosomal abnormality (Table 2). HRAS mutations appear to be relatively common in Spitz nevi and rare in atypical Spitz tumors and Spitz melanomas. Moreover, in these latter tumors, PTEN mutations and heterozygous loss of 9p21 may occur. Spitz melanomas may show TERT promoter mutations [42].

Many studies are currently in progress. Next-generation-sequencing assays may be beneficial in improving interobserver agreement of challenging Spitz neoplasms and improve diagnosis and prognostication of these tumors [47,48]. Hopefully, future investigations will provide better understanding of Spitz tumors, suggesting new, more efficient approaches for this category of controversial tumors.

## Figures and Tables

**Figure 1:**
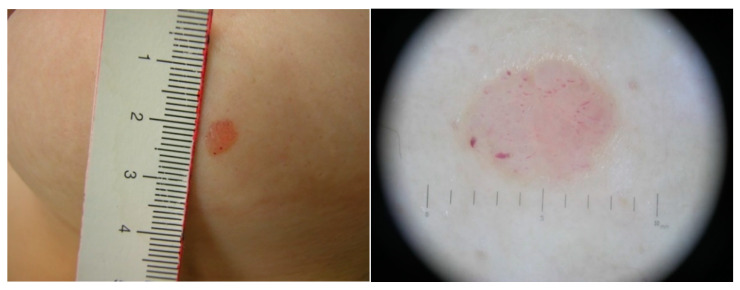

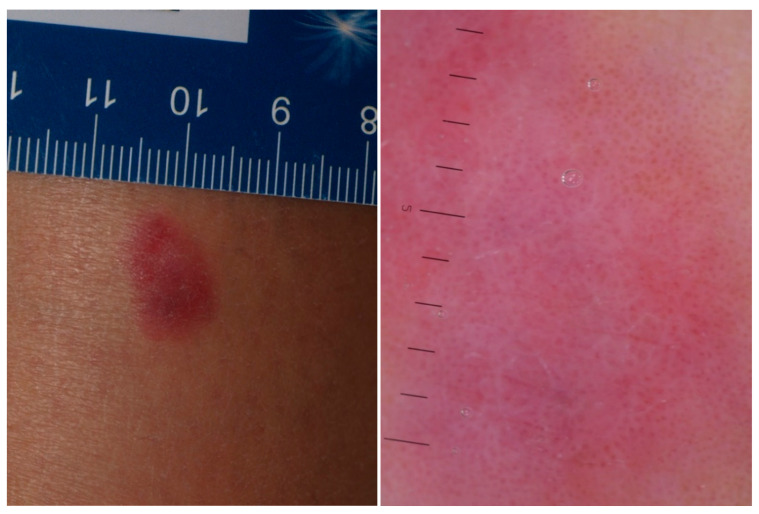
Spitz nevus in a 35-year-old female (upper row): (**left**) Red dome-shaped papule (6 × 5 mm) on the mammary region; (**right**) Dermoscopy shows a featureless pink papule with polymorphous vessels admixed with crystalline structures. Atypical Spitz Tumors in a 23-year-old female (lower row): (**left**) Amelanotic macule (12 × 6 mm) of the lower limb; (**right**) Dermoscopy shows the presence of glomerular vessels uniformly distributed within the entire lesion, without asymmetry, with central reticular depigmentation.

**Figure 2:**
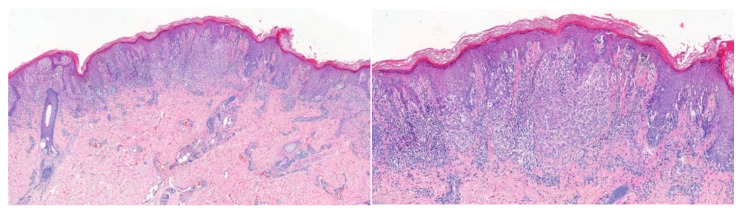
2-year-old male, left buttock–Atypical Spitz Tumor; **left**: compound atypical melanocytic proliferation composed of spindle and epithelioid melanocytes; **right**: confluent irregular nests with solid growth of melanocytes associated with a brisk lymphocytic infiltrate.

**Figure 3:**
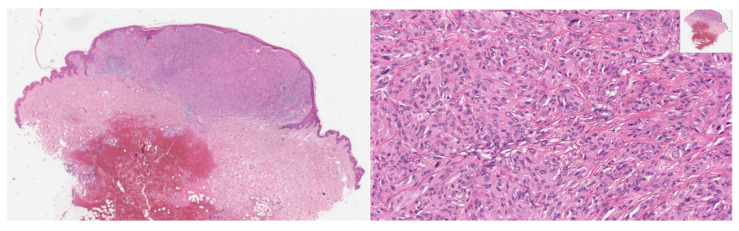

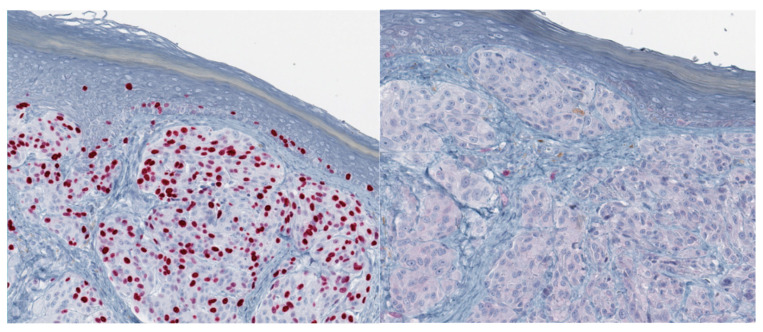
43-year-old female, abdomen–Spitz melanoma; upper row: asymmetric melanocytic proliferation composed of enlarged, nodular or sheet-like aggregates of melanocytes and diminished maturation; spindle and epithelioid melanocytes show nuclear enlargement, pleomorphism, thickening and irregularity of nuclear membranes and prominent nucleoli; lower row: high proliferative activity by Ki-67/MIB-1 (**left**) and loss of p16 by immunohistochemistry (**right**).

**Table 1:** Main genetic alterations in Spitz tumors and other melanocytic lesions.

Spitz Tumors	HRAS Mutation; MAP2K1 Mutation; ALK, ROS1, NTRK1, NTRK3, MAP3K8, BRAF, RET, MET, NTRK2, ERB4, FGFR1, MAP3K3, PRKDC Kinase Fusions
Conventional nevi and melanomas	BRAF/NRAS mutations
Blue tumors	GNAQ/GNA11 mutations
Deep penetrating tumors	BRAF mutation + CTNNB1 mutation or APC loss
Pigmented epithelioid melanocytomas	BRAF mutation + PRKAR1A mutation or PRKCA fusion
BAP1-inactivated tumors	BRAF mutation + BAP1 mutations

**Table 2:** Main differential molecular features in Spitz tumors.

Spitz Nevi	CGH: Isolated Gains 7p and 11p; Tetraploidy; HRAS Mutation
Atypical Spitz tumors	CGH: ≥1 chromosomal abnormality; PTEN mutation; heterozygous loss of 9p21
Spitz melanoma	CGH: ≥1 chromosomal abnormality; PTEN mutation; heterozygous loss of 9p21; TERT promoter mutations
